# Network Pharmacology Study on the Mechanism of Gastrodin Reversing Depressive Symptoms in Traumatically Stressed Rats

**DOI:** 10.2174/1386207325666220928143206

**Published:** 2023-04-27

**Authors:** Ruodan Zhao, Xie Li, Haizhu Zhang, Xubing Chen, Ying Wang

**Affiliations:** 1 School of Pharmacy, Dali University, Dali 671000, China

**Keywords:** Network pharmacology, gastrodin, antidepressant, neuropharmacology, neuron, depression

## Abstract

**Background:**

Depression is a typical outcome of the repair of posttraumatic stress disorder (PTSD). Based on network pharmacology and neuropharmacology experiments, this study aimed to explore how gastrodin (GAS) reverses depressive symptoms in traumatically stressed rats.

**Methods:**

GAS-related targets were predicted by SwissTargetPrediction; depression-related targets were collected from GeneCards and therapeutic target database (TTD); protein-protein interaction (PPI) network was constructed with its action mechanism being predicted by gene ontology (GO) analysis and Kyoto Encyclopedia of Genes and Genomes (KEGG) enrichment. The animal model of PTSD was replicated by single prolonged stress (SPS). The antidepressant effect of GAS was investigated by the forced swim test (FST) and tail suspension test (TST). The levels of tyrosine hydroxylase (TH) and corticotropin-releasing factor type I receptor (CRF1) in locus ceruleus (LC) and the expression of corticotropin-releasing factor (CRF) in the paraventricular nucleus of the hypothalamus (PVN) and central amygdala (CeA) were measured by immunofluorescence.

**Results:**

GAS significantly shortened the tail suspension and swimming immobility in SPS rats in TST and FST experiments (*p* < 0.05 or *p* < 0.01). The network analysis showed that the critical antidepressant targets of GAS were 86 targets such as GAPDH, CASP3 MMP9, HRAS, DPP4, and TH, which were significantly enriched in the pathways such as pathways neuroactive ligand-receptor interaction. High doses of GAS could significantly reduce the level of TH and CRF in CEA in the brain of rats with depressive symptoms (*p* < 0.01) and, at the same time, lower the expression of CRF in PVN (*p* < 0.05).

**Conclusion:**

The effect of GAS on depressive symptoms in SPS rats may be closely related to its reduction of CRF expression in PVN and CeA and inhibition of neuron (NE) synthesis in LC.

## 1 INTRODUCTION

Posttraumatic stress disorder (PTSD) is a delayed-onset, long-term persistent psychological disorder resulting from exposure to a traumatic event and is characterized by four core symptom clusters: traumatic reexperience, avoiding trauma-related stimuli, emotional numbness, and high arousal. It has been found that 70% of the general population will experience a traumatic event at least once in their lifetime, and approximately 10-20% of individuals who experience trauma will develop PTSD. About 1/3 of these patients do not recover for life, and more than 1/2 have substance abuse and other psychiatric disorders [1]. The long-term persistence of PTSD psychiatric disorders seriously impairs patients' social interaction, work, and quality of life [2, 3].

Antidepressant drugs are currently recognized as first-line treatment for PTSD, such as fluoxetine, sertraline, paroxetine, venlafaxine, *etc*. Although antidepressants improve the core symptoms of PTSD to some extent, they also have many limitations. For example, 20%-30% of patients will experience treatment resistance when treated with such drugs, which means that the effectiveness of antidepressants for PTSD is only 70%-80%. In addition, the adverse reactions of antidepressants are apparent, such as weight gain, decreased sexual function, and other mental disorders [4, 5]. The above-mentioned drawbacks limit the use of antidepressants; thus, developing highly effective drugs with fewer side effects for treating PTSD is one of the current hot spots in drug research.

Chinese medicine has been clinically validated for many years, highlighting its multi-systemic, multi-faceted, and multi-targeted therapeutic advantages [1]. *Gastrodia elata* Blume (GE) is one of China's most famous traditional Chinese herbal medicines. Modern pharmacological studies have shown that GE has central effects such as antianxiety [6] and anti-depression [7, 8]. Both ethyl acetate and ethanol extracts of GE could shorten the immobility time of tail suspension and forced swimming in mice. However, the mechanism of the antidepressant effect of Gastrodin (GAS) is less studied, mainly focusing on its effect on the function of the hypothalamic-pituitary-adrenal (HPA) axis, but the effect on the level of central neurotransmitters is not apparent.

Network pharmacology integrates techniques and contents from multiple disciplines, such as bioinformatics and multidirectional pharmacology, and constructs network analysis from a multi-target research strategy of holistic regulation, whose distinctive feature is the overall systematisms. In this experiment, combined with network pharmacology, the component target disease network of GAS antidepressant and the corresponding target protein-protein interaction (PPI) network were constructed, and the core target of GAS antidepressant was screened. The core of the NE-CRF neural loop was used to explore the mechanism of action of GAS in reversing the depressive symptoms induced by traumatic stress in rats.

## 2 MATERIALS AND METHODS

### 2.1 Test Experimental Drugs and Reagents

GAS injection (Kunming Pharmaceutical Group Co., Ltd., batch number: 17EX07-112, Specifications: 0.2g/2ml); Positive drug fluoxetine hydrochloride (PATHEON FRANCE, batch number: 7320B, specification: 20 mg /tablet); Primary antibody:Mouse anti tyrosine hydroxylase (TH), (1:1000, sigma, catalog #T1299); Goat anti-CRFR, (1:500, Abcam, catalog #ab59023); Rabbit anti-CRF, (1:500, peninsula laboratories international, catalog #T-4037.0050). Second antibody: Alexa Fluor 488 donkey anti-mouse IgG antibody (1:500, Jackson Immuno Research Laboratories, catalog #715-546-150); Cy3 conjugated donkey anti-goat IgG antibody (1:500, Jackson Immuno Research Laboratories catalog #705-166-147); Alexa Fluor 488 donkey anti-rabbit IgG antibody (1:500, Jackson Immuno Research Laboratories, catalog #711-546-152).

### 2.2 Laboratory Apparatus

Frozen slicer (Thermo Scientific, model: HM525); Cryogenic tissue slicer (Leica, model: CM1950); Fluorescence quantitative polymerase chain reaction (PCR) instrument (Roche, model: Light Cycler 480); Probe *in vivo* confocal microscope (Nikon, model: NiE-A1plus); Binocular fluorescence/microscope (Leica, model: DM4000B); Analytical balance (Mettler-Toledo instrument Shanghai Co., Ltd., model: MS204S); Constant temperature water bath (Jintan Hualong Experimental instrument Factory, HH-S8); High-speed cryogenic centrifuge (TOMOS limited company,model:3-18R); Glass slide (Fisher Scientific, catalog #12-550-15).

### 2.3 Experimental Animal

Male Sprague Dawley (SD) rats weighing 250 ± 20 g (provided by the Experimental Animal Center of Dali University, license number: P201700619).

### 2.4 Animal Feeding Environment

Rats were housed in a bright and dark light environment (12 hours lighting / 12 hours darkness, illumination time: 7:00-19:00), the indoor temperature was maintained at 24°C ± 2°C, the relative humidity was 45%-60%, the noise was less than 40dB, and the daytime light intensity was controlled at about 300lux. The feeding and water supplementation time for experimental animals were fixed between 18:00 and 18:30 daily.

### 2.5 Database and Software

PubChem database (https://pubchem.ncbi.nlm.nih.gov/), Swiss Target Prediction database (http://www.Wisstargetprediction.ch), GeneCards database (https://www.gene cards.org/), therapeutic target database (TTD) database (http://bigd.big.ac cn/database commons/search). Venny 2.1 software (https://bioinfogp.cnb.csic.es/tools/venny/), Cytoscape 3.6.1 software, STRING database (https://string-db.org/), the database for annotation, visualization and integrated discovery (DAVID) database (https://david.ncifcrf.gov/) and so on.

### 2.6 Experimental Animal Grouping

The experimental animals were randomly divided into sham operation group (n=10), model group (n=10), positive-drug group (n=10), high-GAS group (n=10), and low-GAS group (n=10). The high and low doses of GAS were 0.2 g/kg and 0.1 g/kg, and the dose of fluoxetine hydrochloride was administered at a dose of 0.01 g/kg. The blank control and model groups were given the corresponding volume of distilled water. The therapeutic drugs were given from the next day of SPS stress for 7 consecutive days.

### 2.7 Replication of PTSD Animal Model

According to the SPS established by Liberzon *et al.* [9], the animal model of PTSD was replicated: each group of experimental animals was fixed in a conical glass fixator for 2 h, and then five experimental animals were immediately placed in a plastic drum (55.6 cm in diameter and 45.4 cm in height) with water up to 2/3 of the height of the barrel. And the water temperature was maintained at 24 ± 1°C) for forced swimming for 20 min. After resting for 15 min, the experimental animals were placed in a closed glass desiccator, anesthetized until they lost consciousness, and then removed from the desiccator and placed in a separate ventilated cage without disturbance for 1 week before conducting behavioral experiments.

### 2.8 Forced Swim Test

A forced swim test was performed by forced swim test after the completion of modeling as described by Porsolt. After 24 hours of pre-swimming (15min), the rats were placed in a transparent plastic bucket (water depth above 30 cm, water temperature 23°C) for 5 min, and a camera recorded their behaviors during this period. According to the swimming behavior of the rats, they were divided into three states: floating, struggling, and swimming. Among them, the floating immobility of the rats was defined as the animal stopped struggling in the water and was floating, with only small movements to keep the head afloat, and the disappearance of struggling behavior for 3 seconds was considered as immobility. The learned helplessness was judged by the duration of floating immobility of the rats.

### 2.9 Tail Suspension Test

The rats were suspended from the end of the tail at a distance of 1.5 cm with adhesive tape so that their heads were facing downward, 50 cm from the ground, and the rats showed passive suspension and were considered immobile when the limb movement disappeared. The rats were suspended for 6 minutes, and the cumulative immobility time was recorded for the next 4 minutes [10].

### 2.10 Network Pharmacological Analysis

#### 2.10.1 Screening of Drug Targets

The chemical constituent GAS was submitted to the PubChem database, and its 2D structure (*.SDF) was downloaded. The obtained 2D structure was submitted to the Swiss Target Prediction database, the Homo sapiens were selected as the target species, and the rest parameters were kept as default. As well as collecting the targets of GAS through Chinese and foreign literature research to build the GAS target dataset.

#### 2.10.2 Collection of Disease Targets

In GeneCards and TTD databases, Homo sapiens was used as a qualifier to search, and “antidepressant, depression, posttraumatic stress disorder, depressive, depressed, depression for depressive symptoms analysis” as keywords were searched to collect antidepressant targets.

#### 2.10.3 Network Construction and Analysis

The intersection information between the targets of GAS and the disease targets was submitted to Venny 2.1 software to obtain the potential targets. The potential targets and GAS were introduced into Cytoscape 3.6.1 software to construct the “component-target-disease” interaction network. We input the potential target information into the STRING database, constructed a PPI network, and imported it into Cytoscape 3.6.1 software for visualization.

#### 2.10.4 KEGG Pathway Enrichment and GO Analysis

To further explore the biological functions of potential targets, the network constructed by “Network construction and analysis” was imported into the DAVID database for KEGG pathway enrichment and GO analysis. The selection identifier of the functional annotation tool was set to “UNIPORT_ACCESSION,” the list type was selected as “Gene list,” and the species was limited to “Homo sapiens”. The enrichment analysis of the KEGG signal pathway and GO biological process with *p* < 0.05 were screened out, and the Bioinformatics platform was adopted to plot the advanced bubble diagram.

### 2.11 Animal Experiment Verification

#### 2.11.1 Take Material

1.5 hours after the end of the behavioral experiment, 5 rats in each group were randomly selected and immediately anesthetized with sodium pentobarbital (40 mg/kg) intraperitoneally. The brain tissue was fixed by cardiac perfusion of 4% paraformaldehyde, stripped out of the whole brain, fixed after 4% paraformaldehyde at 4°C and left overnight. The brain tissue was put into 5%, 10%, and 20% sucrose solution for gradient dehydration. The processed brain tissue was put into an optimum cutting temperature (OCT) embedding agent; a frozen sectioning machine performed coronal sections. Locus coeruleus (bregma-9.20~-10.04 mm), central amygdaloid nucleus (bregma-1.80~-3.00 mm), and hypothalamic paraventricular nucleus (bregma-1.20~-1.92 mm) were located with reference to the rat brain stereotaxic atlas, and the brain areas containing the above. The slices containing the above brain regions were attached to slides and then stored at -20°C for fluorescence immunohistochemical detection.

#### 2.11.2 Fluorescence Immunohistochemistry

The prepared rat brain slices were baked at 50°C for 15min, placed in a mailer, rinsed with phosphate-buffered solution (PBS), and sealed with a blocking solution for 1 hour. The sections were incubated with primary antibody at 4°C overnight, rinsed with PBS, and incubated with secondary antibody for 1 h. The sections were sealed with a Mowoil blocker, and pictures were collected by confocal microscopy.

## 3 RESULT

### 3.1 The Effect of GAS on Swimming Immobility Time and Tail Suspension Immobility Time in Rats

**Fig. (1):**
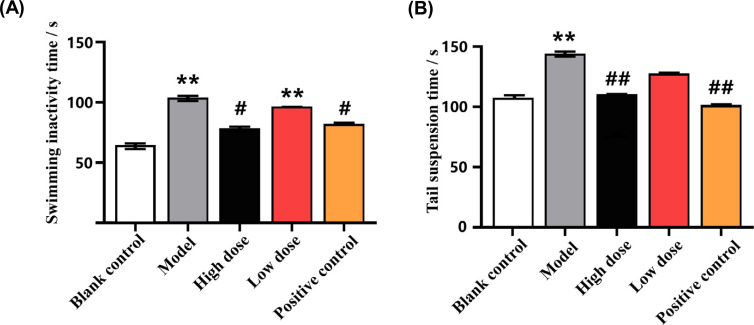
The effect of GAS on immobility time of forced swimming and tail suspension in SPS rats. (**Note:** Fig. (**A**): results of forced swimming experiment; Fig. (**B**): results of tail suspension experiment. Compared with the blank control group, *p < 0.05, **p < 0.01; Compared with the model group, ^#^*p* < 0.05, ^##^*p* < 0.01).

In the forced swimming experiment, compared with the blank control group, the immobility time of rats in the model group was significantly increased (*p* < 0.01). Compared with the model group, high-dose GAS and positive drug fluoxetine hydrochloride significantly reduced the swimming immobility time of rats (*p* < 0.05), while the effect of low-dose GAS was not noticeable (Table **1** and Fig. **1**).

In the tail suspension test, the tail suspension time of the model group was significantly longer than that of the blank control group (*p* < 0.01). High-dose GAS and positive drug fluoxetine hydrochloride significantly reduced the tail suspension time of rats (*p* < 0.01), while the effect of low-dose GAS was not noticeable (Table **1** and Fig. **1**).

### 3.2 Network Pharmacological Analysis

#### 3.2.1 Potential Target Screening

A total of 101 GAS targets were collected through literature [9] investigation and target prediction. A total of 13,338 disease targets were obtained by GeneCards and TTD database, and 93 potential action targets were obtained after taking the intersection with the action targets of the chemical component GAS.

#### 3.2.2 The Network Structure of “Component-Target-Disease”

**Fig. (2):**
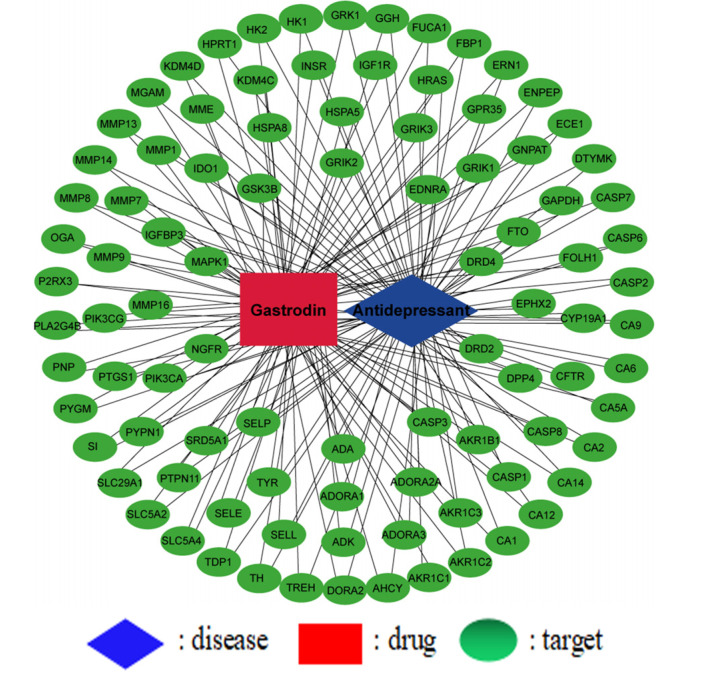
Chemical composition potential target interaction network.

The information on GAS and 93 potential targets were introduced into Cytoscape 3.6.1 software to construct the “component- target-disease” network and draw the network map (Fig. **2**).

#### 3.2.3 PPI Network Architecture

**Fig. (3):**
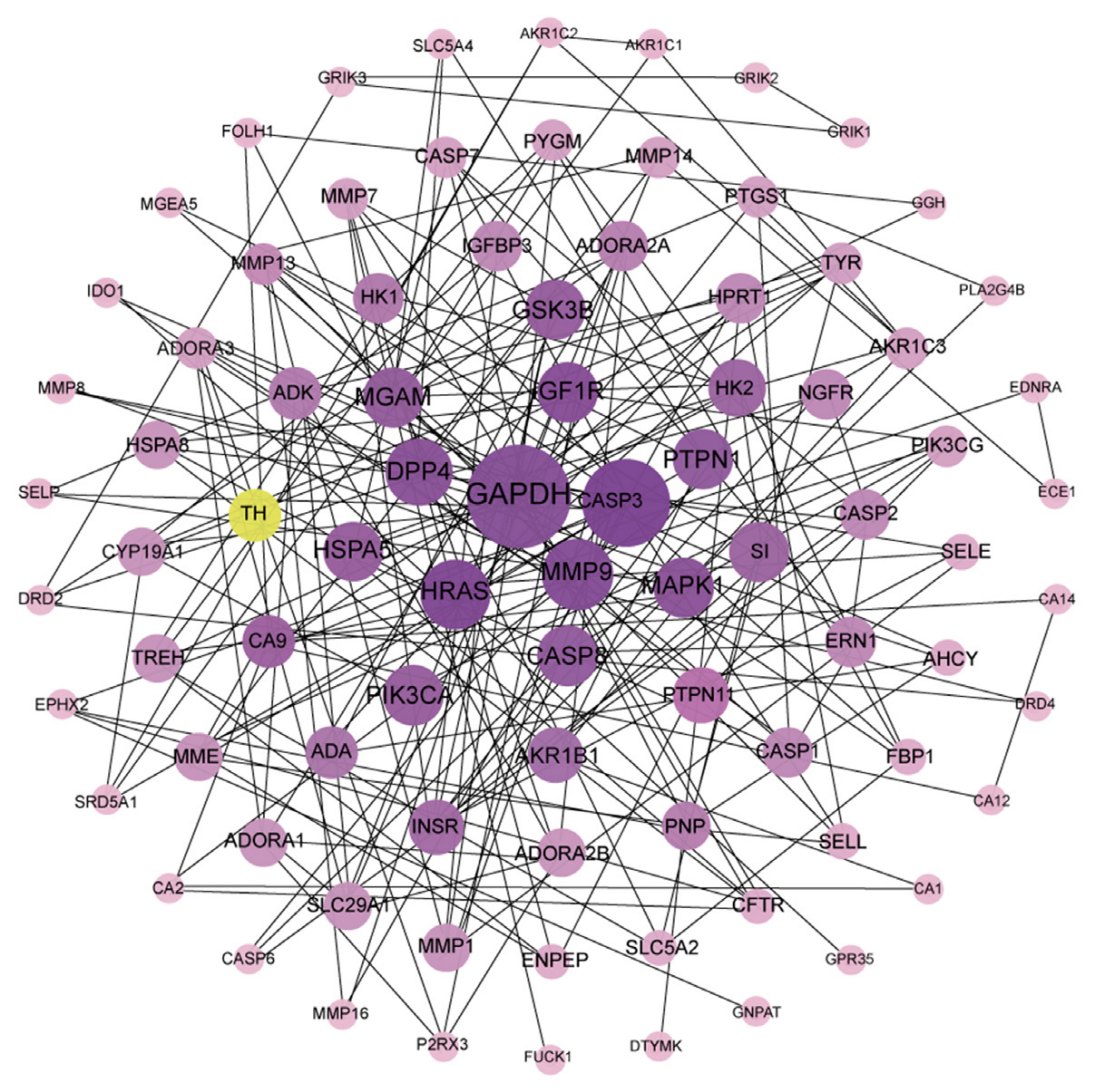
Protein-protein interaction network. **Note:** The size and color depth of the node is proportional to the node degree value.

The potential targets were imported into the STRING database to obtain the protein-protein interaction data and visualized by Cytoscape software to construct the PPI network (Fig. **3**), which contains 86 nodes and 319 edges after topological analysis. TH targets interact with 9 targets, including ADORA2A, AKR1B1, CASP3, DRD2, DRD4 and GAPDH.

#### 3.2.4 Enrichment of KEGG Path and Bioinformatics Analysis of GO Compounds

**Fig. (4):**
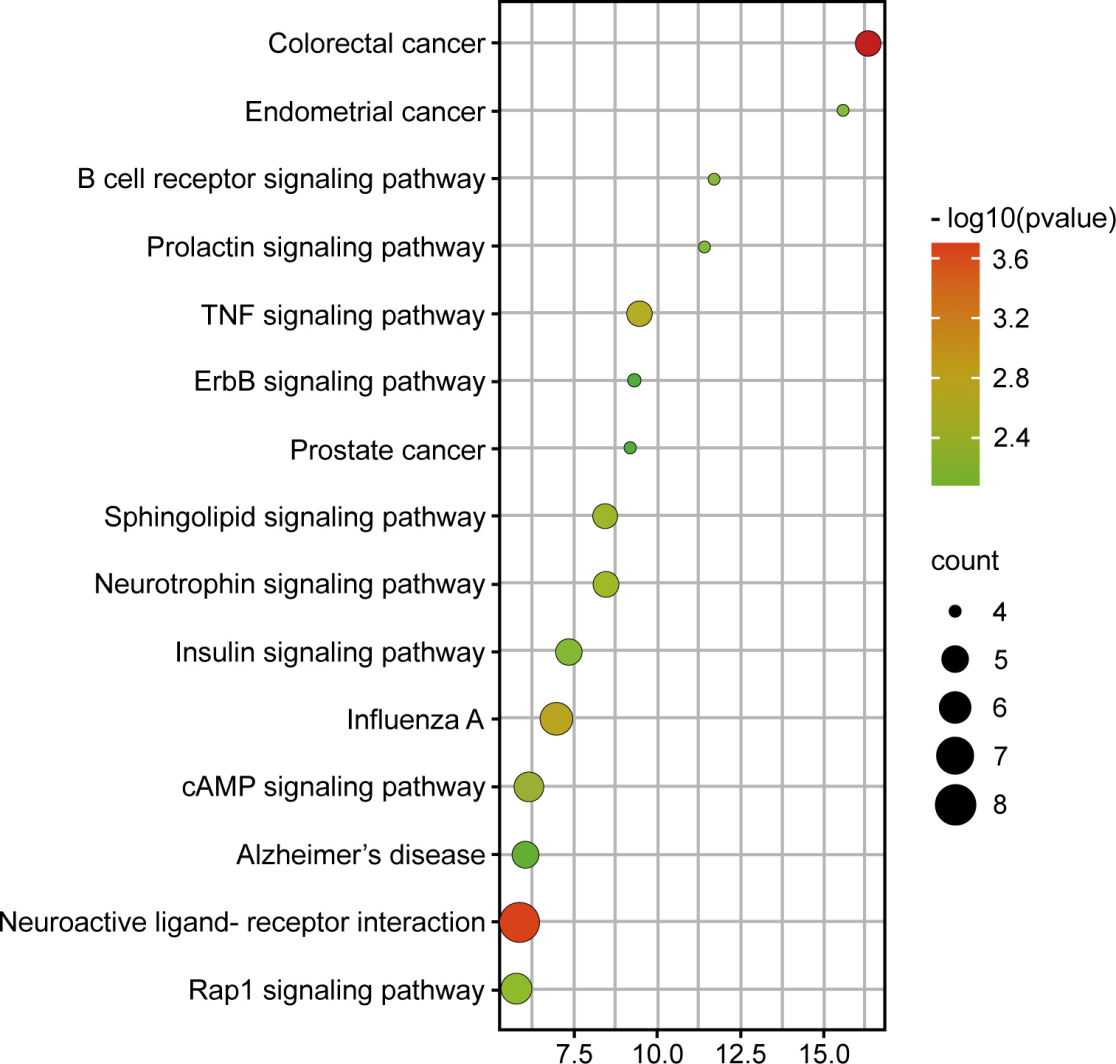
KEGG pathway enrichment analysis.

**Fig. (5):**
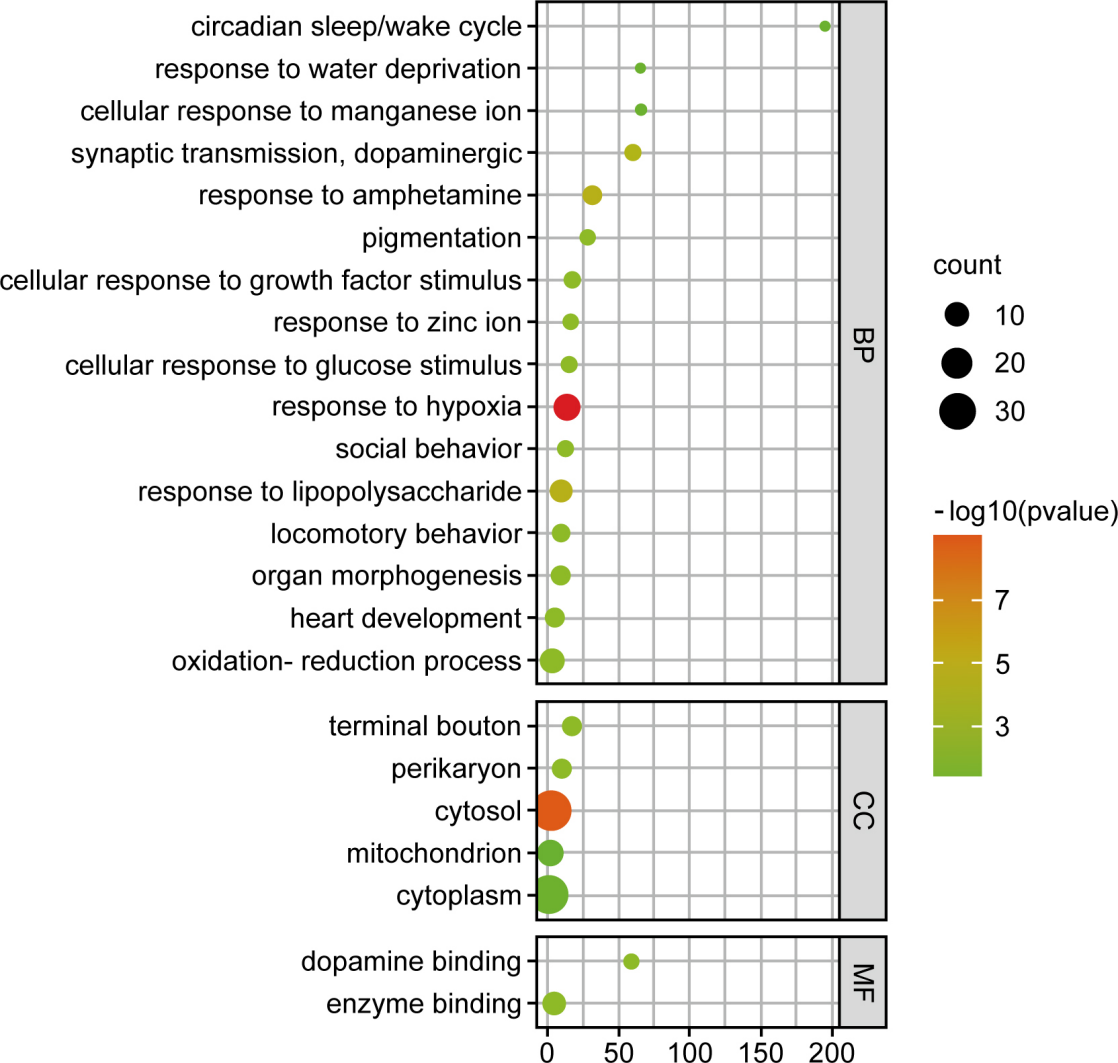
GO bioinformatics analysis.

Sixty-one signal pathways were obtained by introducing the potential targets into the DAVID database, mainly related to neuroactive ligand-receptor interaction, colorectal cancer, influenza A, TNF signaling pathway, cAMP signaling pathway, Rap1 signaling pathway, and so on (Fig. **4**). Among them, the neuroactive ligand-receptor interaction was highly relevant, containing 13 gene targets, including EDNRA, P2RX3, ADORA2A, ADORA2B, TH, GPR35, ADORA3, ADORA1, GRIK1, GRIK2, GRIK3, DRD2, and DRD4. GO bioinformatics analysis yielded 204 GO entries, of which 115 biological processes (BP) entries, 33 cell composition (CC) entries, and 56 molecular functions (MF) entries (Fig. **5**). The BP mainly involved the response to hypoxia, the oxidation-reduction process, the response to lipopolysaccharide, *etc*. CC involved many components, such as cytosol, cytoplasm, mitochondrion, terminal bouton, etc. Furthermore, the MF mainly involved dopamine binding, enzyme binding, *etc*.

### 3.3 Animal Experiment Verification

#### 3.3.1 Effects of GAS on the Expression of CRF, CRFR1, and TH in Rat Brain

**Fig. (6):**
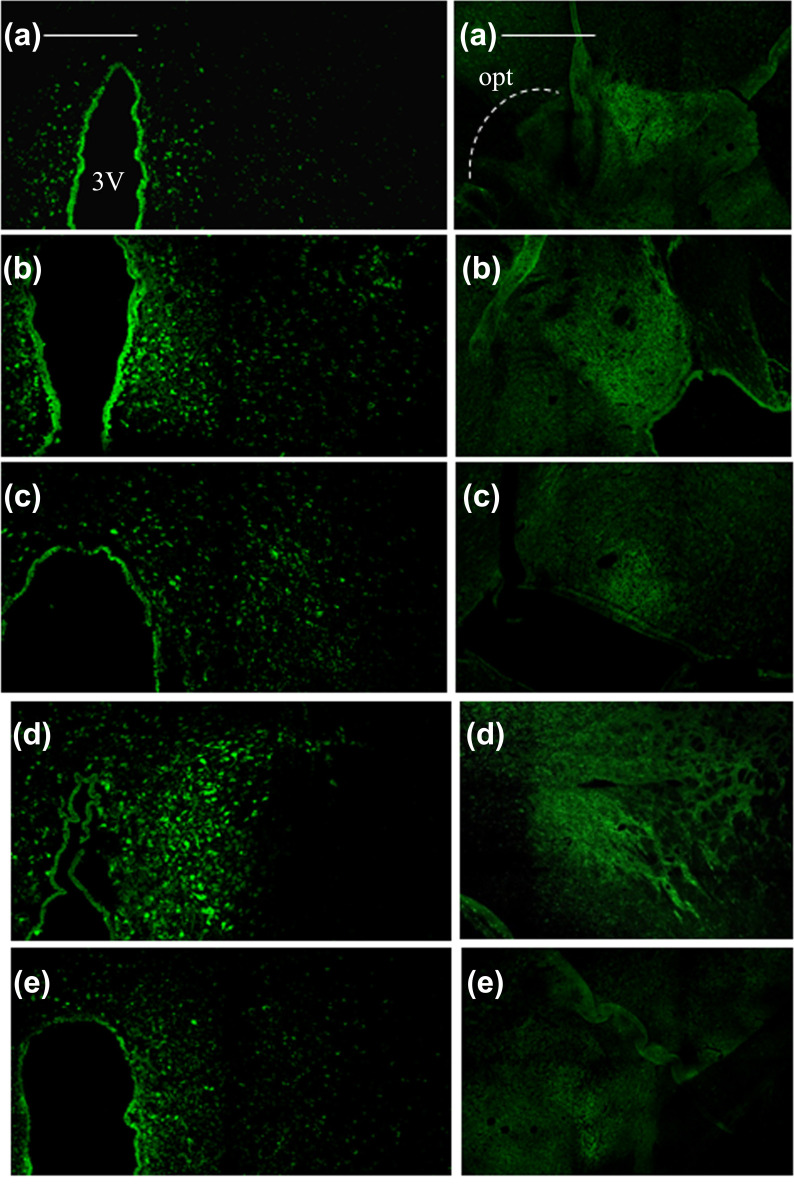
The effect of GAS on the expression of CRF protein in the PVA and CeA. (The left image column represents the CRF protein expression in PVN. The right column of images represents the expression of CRF protein in CeA. 3V: The third ventricle; **a**: Blank control group, **b**: Model group, **c**: High dose GAS group, **d**: Low dose GAS group, **e**: Positive drug group. Scale: 50 μm, Z-stack: 10-layer overlay).

**Fig. (7):**
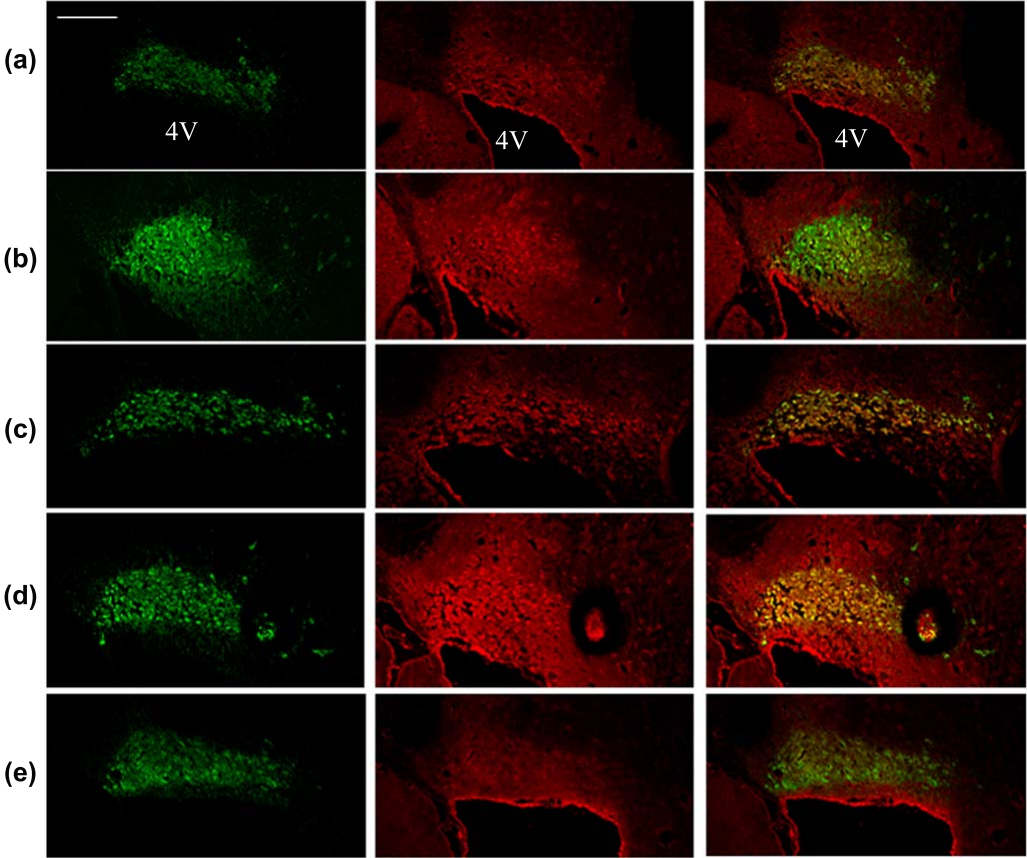
The effect of GAS on the expression of TH and CRFR1 protein in locus ceruleus. (Green fluorescence: TH protein expression; the red fluorescence: CRFR1 protein expression, 4V: Fourth ventricle, **a**: Blank control group, **b**: Model group, **c**: High dose GAS group, **d**: Low dose GAS group, **e**: Positive drug group. Scale: 50 μm, Z-stack: 13-layer overlay).

**Fig. (8):**
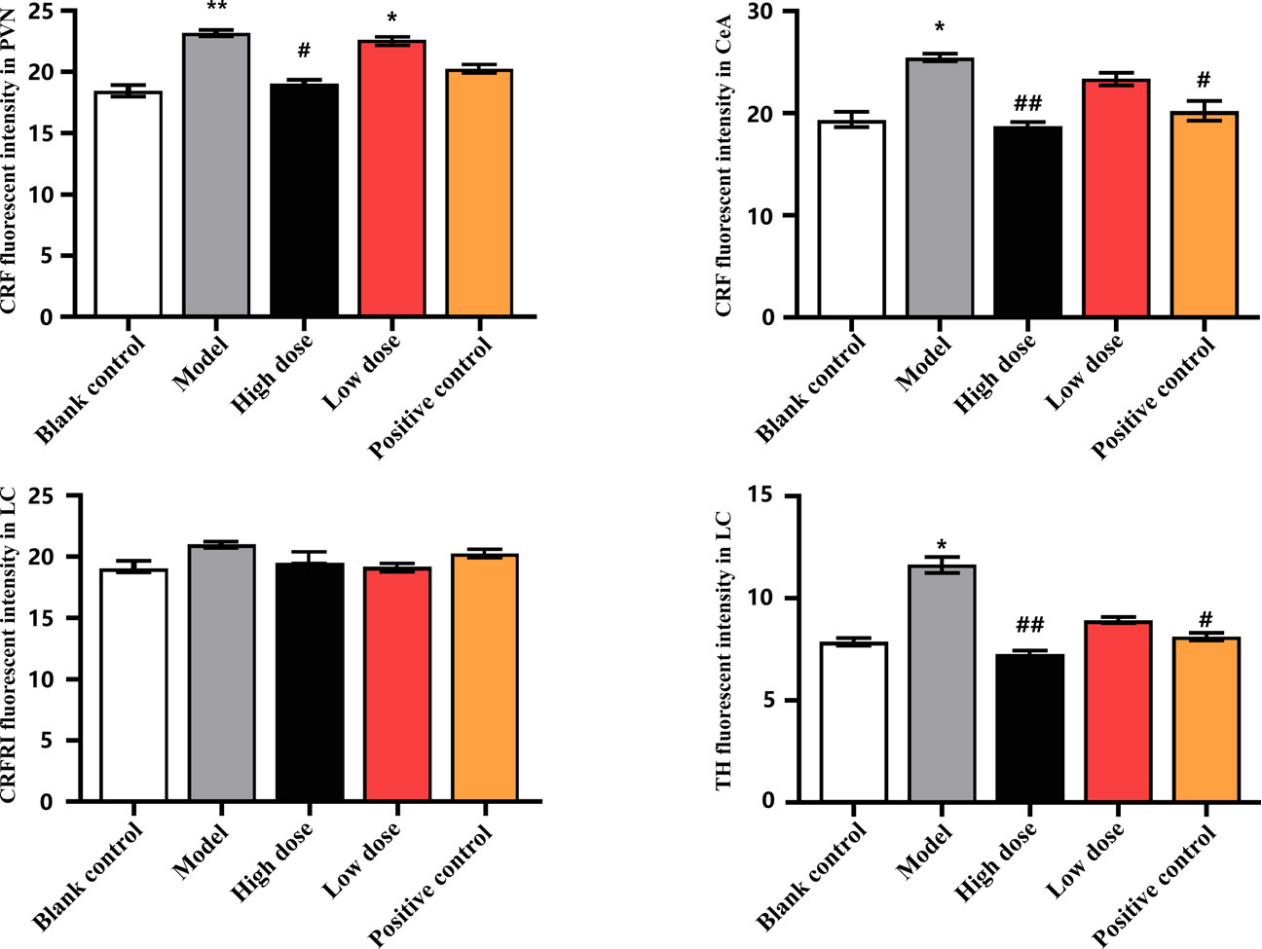
The effect of GAS on CRF, CRFR1 and TH (**Note**: compared with the blank control group, *p < 0.05; compared with the model group, ^#^*p* < 0.05; *p* < 0.01).

Compared with the blank control group, the expression of CRF in the paraventricular nucleus (PVN) and central amygdala (CeA) of the model group was significantly increased (*p* < 0.01 and *p* < 0.05) (Table **2** and Fig. **6**), and the expression of tyrosine hydroxylase (TH) and in locus coeruleus (LC) was significantly enhanced (*p* < 0.05) (Table **2** and Fig. **7**). Compared with the model group, fluoxetine hydrochloride significantly decreased the level of TH in LC (*p* < 0.05) and the expression of CRF in CeA (*p* < 0.05); High dose GAS significantly decreased CRF levels within TH and CEA (*p* < 0.01), and CRF expression in PVN (*p* < 0.05) (Table **2** and Fig. **8**).

## 4 DISCUSSION

The depressive symptoms accompanying PTSD have more complex pathogenesis than simple depression. In addition to the involvement of the peripheral HPA axis, there are also dysfunctions of the intra-central nervous system, most notably CRF, which is not only involved in regulating the function of the HPA as a hypothalamic hormone but also in the central amygdala, where it is independently involved in the development of the PTSD course as a neurotransmitter [11]. On the other hand, there are direct and indirect nerve projections between the CRF nervous system and the central NE nervous system, and both may play a joint role in the disease process of PTSD [12-14].

In this study, the potential targets and mechanisms of antidepressant effects of GAS were predicted by network pharmacology, and potential targets such as GAPDH, CASP3, and TH were identified. The antidepressant effects were mainly achieved through neuroactive ligand-receptor interaction, TNF signal pathway, and cAMP signal pathway. The correlation between neuroactive ligand-receptor interaction is the most significant among them, which collects all receptors and ligands related to intracellular and extracellular signal pathways on the plasma membrane and participates in transmitting neurotransmitters [15]. This pathway is significantly enriched in other antidepressants in traditional Chinese medicine and compounds. It is speculated that this pathway may also play a vital role in the antidepressant process of GAS. Moreover, there is evidence that GAS can improve the depressive symptoms of PTSD rats, which is related to the reduction of NE in the hippocampus and TH, the rate-limiting enzyme of NE synthesis in the blue shift LC. It has been suggested that the depressive symptoms associated with PTSD may be related to the dysfunction of the NE nervous system in the center, but the brain regions and nuclei associated with LC-NE nerves are not mentioned.

Given the anatomical and functional relationship between NE and CRF nerves, combined with the disease targets predicted by network pharmacology, this experiment provides an in-depth analysis of the mechanisms of GAS for depressive symptoms in PTSD, with the CRF-NE nerve loop as the core. The results showed that a high dose of GAS (0.2 g/kg) significantly shortened the tail suspension and swimming time of SPS rats in TST and FST, suggesting an excellent antidepressant effect, and its antidepressant effect was similar to that of fluoxetine hydrochloride, a positive control drug, which was consistent with those reported by BombiLee *et al*. Animal experiments showed that GAS could inhibit the synthesis of tyrosine hydroxylase in LC, thus reducing the synthesis of NE in LC. At the same time, double fluorescence immunostaining images showed many CRFR1 receptors in the LC, indicating that there were CRF afferents to nerve fibers in the LC. CRF nerve fibers in the CeA nucleus [16] and hypothalamic PVN [17] have been reported to project directly to LC and act on CRFR1 receptors to excite LC. It is speculated that the CRF nerve may be the upstream mechanism of LC excitation.

Furthermore, we determined the number of CRFR1 receptors in LC, indicating GAS had no effect on the number of receptors but could significantly reduce the expression of CRF in PVN and CeA, which proved that GAS improved the depressive symptoms of SPS rats by reducing the level of CRF in PVN and CeA, and thus attenuating the excitatory effect of CRF nerves on LC. CRF may be the upstream mechanism of LC-NE. GAS showed a broader central effect and affected CRF levels in PVN and CeA compared with positive drugs, which only decreased CRF levels in CeA. In addition, we found elevated CRF levels in the CNS of SPS rats, similar to the depression-only model. After the GAS intervention, the level of CRF decreased, and the depressive symptoms of rats improved. The results suggest that the depressive symptoms of SPS rats intersect with simple depression, showing an increase in CRF level but not the same or even opposite changes in the NE nervous system. NE levels were elevated in PTSD patients [18], while depressed patients had reduced or no change in NE levels [19].

## CONCLUSION

To sum up, GAS has an excellent therapeutic effect on depression associated with PTSD. Its mechanism is to downregulate the level of CRF and then impair the neural function of its downstream target LC-NE to improve depressive symptoms in PTSD. Although the rate-limiting enzymes for NE synthesis have been identified, NE levels and post-receptor effects of CRFR1 are not directly monitored. We will conduct in-depth research on the above issues in the future.

## Figures and Tables

**Table 1:** The effects of GAS on immobility time of forced swimming and tail suspension in SPS rats (X±S, n=10).

**Group**	**Dosage**	**Swimming Inactivity Time/s**	**Tail Suspension Time/s**
Blank control group	-	64.3±12.6	103.5±19.1
Model group	-	103.3±22.1**	138.3±15.3**
High dose GAS group	0.2 g/kg	78.3±13.3^#^	105.9±25.5^##^
Low dose GAS group	0.1 g/kg	95.9±15.2**	122.3±17.1
Fluoxetine hydrochloride	0.01 g/kg	82.3±17.0^#^	97.4±21.4^##^

**Note:** Compared with the blank control group,*p < 0.05,**p < 0.01; Compared with the model group,^#^*p* < 0.05,^##^*p* < 0.01.

**Table 2:** The effect of GAS on CRF, CRF1 and TH (X±S, n=5).

**Group**	**CRF (PVN)**	**CRF(CeA)**	**CRFR_1_**	**TH**
Blank control group	18.39±1.62	19.36±2.34	19.13±3.44	7.92±1.22
Model group	23.16±2.40**	25.39±3.55*	21.15±2.81	11.66±2.14*
High dose GAS group	19.03±1.84^#^	18.68±2.42^##^	19.63±2.12	7.34±1.26^##^
Low dose GAS group	22.21±1.24*	23.34±2.68	19.33±2.76	8.95±1.24
Fluoxetine hydrochloride group	20.24±2.21	20.14±2.67^#^	20.47±3.03	8.13±2.03^#^

**Note:** Compared with the blank control group, *p < 0.05, **p < 0.01; Compared with the model group, ^#^*p* < 0.05, ^##^*p* < 0.01.

## Data Availability

The authors confirm that the data supporting the findings of this research are available within the article.

## LIST OF ABBREVIATIONS

BP: Biological Processes
CC: Cell Composition
CeA: Central Amygdala
CRF: Corticotropin Releasing Factor
CRF1: Corticotropin-Releasing Factor Type I Receptor
DAVID: Database for Annotation, Visualization and Integrated Discovery
FST: Forced Swim Test
GAS: Gastrodin
GE: *Gastrodia elata* Blume
GO: Gene Ontology
HPA: Hypothalamic Pituitary Adrenal
KEGG: Kyoto Encyclopedia of Genes and Genomes
LC: Locus Ceruleus
MF: Molecular Functions
NE: Neurons
OCT: Optimum Cutting Temperature
PBS: Phosphate Buffered Solution
PCR: Polymerase Chain Reaction
PPI: Protein-Protein Interaction
PTSD: Posttraumatic Stress Disorder
PVN: Paraventricular Nucleus of the Hypothalamus
SD: Sprague-Dawley
SPS: Single Prolonged Stress
TH: Tyrosine Hydroxylase
TST: Tail Suspension Test
TTD: Therapeutic Target Database

## References

[1] Zhang J., Xue R., Zhang Y.Z., Qiu J.Q., Wei H.W. (2021). Effects of exercise on monoamine transmitters and inflammation in rats with post-traumatic stress disorder.. J. Neuoranat..

[2] Avecillas C.J.M., Justo M., Levinson S., Koek R., Krahl S.E., Chen J.W.Y., Lee S.J., Langevin J.P., Bari A. (2020). Structural correlates of emotional response to electrical stimulation of the amygdala in subjects with PTSD.. Brain Stimul..

[3] Deng M.Y. (2016). New progress in clinical research of posttraumatic stress disorder (DSM-5 new standard).. Chinese J. Health Psychol..

[4] Hoskins M., Pearce J., Bethell A., Dankova L., Barbui C., Tol W.A., Van Ommeren M., De Jong J., Seedat S., Chen H., Bisson J.I. (2015). Pharmacotherapy for post-traumatic stress disorder: Systematic review and meta-analysis.. Br. J. Psychiatry.

[5] Stein D.J., Ipser J., McAnda N. (2009). Pharmacotherapy of posttraumatic stress disorder: A review of meta-analyses and treatment guidelines.. CNS Spectr..

[6] Peng Z., Wang H., Zhang R., Chen Y., Xue F., Nie H., Chen Y., Wu D., Wang Y., Wang H., Tan Q. (2013). Gastrodin ameliorates anxiety-like behaviors and inhibits IL-1beta level and p38 MAPK phosphorylation of hippocampus in the rat model of posttraumatic stress disorder.. Physiol. Res..

[7] Zhang R., Peng Z., Wang H., Xue F., Chen Y., Wang Y., Wang H., Tan Q. (2014). Gastrodin ameliorates depressive-like behaviors and up-regulates the expression of BDNF in the hippocampus and hippocampal-derived astrocyte of rats.. Neurochem. Res..

[8] Lee B., Sur B., Yeom M., Shim I., Lee H., Hahm D.H. (2016). Gastrodin reversed the traumatic stress-induced depressed-like symptoms in rats.. J. Nat. Med..

[9] Liberzon I., López J.F., Flagel S.B., Vázquez D.M., Young E.A. (1999). Differential regulation of hippocampal glucocorticoid receptors mRNA and fast feedback: Relevance to post-traumatic stress disorder.. J. Neuroendocrinol..

[10] Steru L., Chermat R., Thierry B., Simon P. (1985). The tail suspension test: A new method for screening antidepressants in mice.. Psychopharmacology.

[11] Sanford C.A., Soden M.E., Baird M.A., Miller S.M., Schulkin J., Palmiter R.D., Clark M., Zweifel L.S. (2017). A central amygdala CRF circuit facilitates learning about weak threats.. Neuron.

[12] Barsegyan A., McGaugh J.L., Roozendaal B. (2014). Noradrenergic activation of the basolateral amygdala modulates the consolidation of object-in-context recognition memory.. Front. Behav. Neurosci..

[13] McGaugh J.L. (2013). Making lasting memories: Remembering the significant.. Proc. Natl. Acad. Sci. USA.

[14] O’Donnell T., Hegadoren K.M., Coupland N.C. (2004). Noradrenergic mechanisms in the pathophysiology of post-traumatic stress disorder.. Neuropsychobiology.

[15] Qiu Y.P., Shen Q.L. (2021). Research on mechanism of Lilii bulbus in treating depression based on network pharmacology.. Anhui Daxue Xuebao.

[16] Van Bockstaele E.J., Colago E.E.O., Valentino R.J. (1996). Corticotropin-releasing factor-containing axon terminals synapse onto catecholamine dendrites and may presynaptically modulate other afferents in the rostral pole of the nucleus locus coeruleus in the rat brain.. J. Comp. Neurol..

[17] Valentino R.J., Page M.E., Curtis A.L. (1991). Activation of noradrenergic locus coeruleus neurons by hemodynamic stress is due to local release of corticotropin-releasing factor.. Brain Res..

[18] Brown R.E., Basheer R., McKenna J.T., Strecker R.E., McCarley R.W. (2012). Control of sleep and wakefulness.. Physiol. Rev..

[19] Shekhar M.S., Venkatachalam T., Sharma C.S., Pratap Singh H., Kalra S., Kumar N. (2019). Computational investigation of binding mechanism of substituted pyrazinones targeting corticotropin releasing factor-1 receptor deliberated for anti-depressant drug design.. J. Biomol. Struct. Dyn..

